# ACE Inhibition Modulates Myeloid Hematopoiesis after Acute Myocardial Infarction and Reduces Cardiac and Vascular Inflammation in Ischemic Heart Failure

**DOI:** 10.3390/antiox10030396

**Published:** 2021-03-05

**Authors:** Wolf-Stephan Rudi, Michael Molitor, Venkata Garlapati, Stefanie Finger, Johannes Wild, Thomas Münzel, Susanne H. Karbach, Philip Wenzel

**Affiliations:** 1 Center for Thrombosis and Haemostasis, University Medical Center Mainz, Langenbeckstraße 1, 55131 Mainz, Germany; stephan.rudi@web.de (W.-S.R.); Michael.Molitor@unimedizin-mainz.de (M.M.); garlapati@uni-mainz.de (V.G.); Stefanie.Finger@unimedizin-mainz.de (S.F.); Johannes.Wild@unimedizin-mainz.de (J.W.); tmuenzel@uni-mainz.de (T.M.); karbasu@uni-mainz.de (S.H.K.); 2 Center for Cardiology-Cardiology I, University Medical Center Mainz, Langenbeckstraße 1, 55131 Mainz, Germany; 3 German Center for Cardiovascular Research (DZHK, Deutsches Zentrum für Herz-Kreislauf-Forschung), Partner Site Rhine-Main, 55131 Mainz, Germany

**Keywords:** myocardial infarction, ACE inhibitors, emergency hematopoiesis, vascular inflammation

## Abstract

Aims: Angiotensin-converting-enzyme inhibitors (ACE inhibitors) are a cornerstone of drug therapy after myocardial infarction (MI) and improve left ventricular function and survival. We aimed to elucidate the impact of early treatment with the ACE inhibitor ramipril on the hematopoietic response after MI, as well as on the chronic systemic and vascular inflammation. *Methods and Results:* In a mouse model of MI, induced by permanent ligation of the left anterior descending artery, immediate initiation of treatment with ramipril (10 mg/k/d via drinking water) reduced cardiac inflammation and the number of circulating inflammatory monocytes, whereas left ventricular function was not altered significantly, respectively. This effect was accompanied by enhanced retention of hematopoietic stem cells, Lin^−^Sca1^−^c-Kit^+^CD34^+^CD16/32^+^ granulocyte–macrophage progenitors (GMP) and Lin^−^Sca1^−^c-Kit^+^CD150^−^CD48^−^ multipotent progenitors (MPP) in the bone marrow, with an upregulation of the niche factors Angiopoetin 1 and Kitl at 7 d post MI. Long-term ACE inhibition for 28 d limited vascular inflammation, particularly the infiltration of Ly6C^high^ monocytes/macrophages, and reduced superoxide formation, resulting in improved endothelial function in mice with ischemic heart failure. *Conclusion:* ACE inhibition modulates the myeloid inflammatory response after MI due to the retention of myeloid precursor cells in their bone marrow reservoir. This results in a reduction in cardiac and vascular inflammation with improvement in survival after MI.

## 1. Introduction

Besides rapid revascularization, medical therapy substantially improves outcomes after acute myocardial infarction (MI). Antiplatelet therapy, ß-blockers, statins and angiotensin-converting-enzyme inhibitors (ACE inhibitors) or angiotensin II receptor type 1 blockers (ARB) form the basis of drug treatment [1].

While the prevention and treatment of cardiovascular risk factors determine current therapeutic strategies, there is growing evidence that MI induces long-lasting changes in the immune system and hematopoiesis, which accelerates inflammation [2,3]. Several immune cells express angiotensin II receptors, primarily type 1, on their surface, which impacts downstream signaling and polarization [4]. Due to phosphorylation of NF-kB, AngII signaling directly influences the levels of inflammatory cytokines such as tumor necrosis factor alpha (TNFα) or interleukin 1 beta (IL1ß), which are drivers of chronic inflammation in cardiovascular diseases [5]. It has been shown that AngII enhances vascular permeability, amplifies the expression of selectins on endothelial cells, platelets and leukocytes and regulates the expression of integrins such as vascular cell adhesion molecule-1 (VCAM-1) and intercellular adhesion molecule-1 (ICAM-1) [6,7,8].

Recently, we could show that circulating myeloid cells drive vascular inflammation in ischemic heart failure. Treatment with the ARB telmisartan for four weeks post-MI improved systemic vascular dysfunction due to local anti-inflammatory effects and diminished bone marrow hematopoiesis in experimental heart failure [9]. There is additional evidence that RAS directly interacts with myeloid hematopoiesis because infusion with AngII caused an accumulation, differentiation as well as expulsion of hematopoietic stem cells (HPSC) out of the bone marrow and spleen [10]. Dutta et al. demonstrated that acute MI induces a severe mobilization of HPSCs, particularly CCR2 ^+^CD150^+^CD48^−^ LSK (lineage-Sca-1^+^ c-kit^+^) cells [3]. Furthermore, MI leads to mobilization of splenic-stored inflammatory Ly6C^high^ monocytes, releasing them into the bloodstream. Mice lacking the angiotensin receptor (Atgr1a^−/−^) showed lower numbers of monocytes released from their splenic reservoir [11].

To close the gap in knowledge between the proven clinical benefits of ACE inhibitor administration and extensively investigated AngII effects, we analyzed the impact of early ramipril treatment after acute MI on the myeloid emergency response in the bone marrow and spleen, as well as the long-term effects on cardiac and vascular inflammation.

## 2. Material and Methods

### 2.1. Chemicals

The chemicals were acquired from Sigma, Merck or Fluka and were of the highest quality and analytical grade.

### 2.2. Ethics Approval

All animal experiments were authorized by the Animal Care and Use Committee of Rhineland-Palatinate. The registries number of approval was G15-1-067, and all experimental procedures conducted conformed to the guidelines from Directive 2010/63/EU of the European Parliament on the protection of animals used for scientific purposes. The housing and treatment were in accordance with the institutional guidelines of the Central Animal Facility of the University Medical Center in Mainz (TARC) and with the relevant laws. Investigators performing animal experiments were in possession of certificates of at least FELASA B (Federation of Laboratory Animal Association) level.

### 2.3. Animals and In Vivo Treatment

Male (8 to 14 weeks old) C57BL/6J mice were purchased from Jackson Laboratory. MI was induced by permanent ligation of the left anterior descending artery (LAD), as described previously [12]. Anesthesia consisted of medetomidine (500 µL/kgBW (body weight)), fentanyl (50 µg/kgBW) and midazolam (5 mg/kgBW) and was injected intraperitoneally before surgical treatment. Antipamezol (2.5 mg/kgBW) and flumazenil (0.5 µg/kgBW) were injected to antagonize anesthesia. After induction of myocardial infarction, mice received buprenorphine (0.075 mg/kgBW) subcutaneously twice a day for a period of two days. Sham procedure was conducted in the same way without ligating the LAD. Treatment with ramipril (10 mg/kgBW/d) started immediately post-MI and was applied via drinking water. We used two different experimental regimes. Cardiac, bone marrow and splenic inflammatory response was analyzed 7 days after myocardial infarction. Additionally, bone marrow response was investigated after 48 h. To investigate long-term effects of ramipril treatment on survival and vascular function, a chronic heart failure model after 28 days was used. Mice were deeply anaesthetized and killed by exsanguination after puncture of the right ventricle. Blood was collected after injection of 200 µL of heparin (1:5); aorta, heart and spleen were dissected carefully and rapidly transferred to 4 °C Krebs-Hepes solution.

### 2.4. Small Animal Echocardiography

High-frequency small-animal ultrasound (HFUS) of the heart was done with a VEVO-770 and 3100 high-resolution imaging system (VisualSonics^®^, FujiFilm, Toronto, CA, USA). We determined the left ventricular function on day 6 after MI. Anesthesia was induced using isoflurane (1.0–1.5 Vol%). Acquisition of echocardiographic images was conducted with a linear array transducer (MZ 400, 38 MHz). During the measurements, breathing and heart rate were normalized due to the anesthetic depth, and body temperature was kept stable. Cineloops of brightness (B) mode and pictures of motion (M) mode in parasternal long axis (PLAX) position were acquired and stored. Left ventricular ejection fraction (LVEF), left ventricular end-diastolic diameter (LVEDD), left ventricular (LV) cardiac output (CO) and stroke volume (SV) were analyzed and computed from B-mode cineloops in PLAX position with the VevoLab Software^®^. Wall motion was scored with 1 for normal, 2 for hypokinetic and 3 for akinetic. WMSI was determined as sum of all scores divided through the amount of evaluated segments (adapted from Zhang et al.) [13].

### 2.5. Flow Cytometry Analysis of Immune Cells

Flow cytometry analysis of heart tissue, blood, bone marrow, spleen and aortic vessels was performed. A fragment of infarcted myocardial tissue was harvested after previous flushing. In SHAM-treated mice, a preferably identical piece of non-infarcted myocardial tissue of the left ventricle was used as a control. The bone marrow was rinsed out of one whole femur and aortic vessels were thoroughly cleared of adhesive tissue.

### 2.6. Processing of Heart Tissue, Bone Marrow, Spleen and Aortic Vessels

Cardiac tissue was mechanically fragmented and then digested by the enzyme collagenase II (1 mg/mL)/DNase I (50 µg/mL) for 30 min at 37 °C. Afterwards, the solution was passed through a cell strainer (70 µm) and diluted with PBS/2% FCS. Spleens were compressed through a cell strainer (40 µm) without further fragmenting and collected in PBS/2% FCS. Flushed bone marrow of one femur was resuspended in PBS/2%FCS and afterwards passed through a 40-µm cell strainer. Aortic vessels were shredded and digested in liberase solution TM (1 mg/mL) at 37 °C for 30 min. Afterwards, the solution was compressed through a 40-µm cell strainer and solved in PBS/2% FCS, as described earlier^12^. ACK lysis buffer was added to whole blood, splenic tissue, bone marrow and heart tissue for 2 to 5 min to dissolve red blood cells.

### 2.7. Cell Staining

Fc-Block (anti-CD16/CD32) was added for preventing unspecific bindings of antibodies. Single cell suspensions of the different tissues (heart, spleen and blood) were stained with subsequent monoclonal antibodies: CD45 (30-F11) in (APC)-eFlour 780, CD90.2 (Thy-1.2) in SB645, NK 1.1 (PK136) in PE-Cy7, F4/80 (BM8) in APC, CD11b (M1/70) in PerCP-Cy5.5, Ly6G (1A8) in PE, Ly6C (Al-21) in Pacific Blue and Viabilty Dye eFlour 506 monoclonal antibodies. The bone marrow was stained with the following antibodies: CD45-APC-eFlour 780 (30-F11), CD11b (M1-70), Ter119 (Ter119), Gr-1 (RB6-8C5), B220 (RA3-6B2), CD19 (eBio1D3), CD8a (53-6-7), CD5 (53-7-3), CD3 (145-2C11), CD2 (RM2-5) all in FITC for lineage gating, CD34 (RAM34) in Pacific Blue, CD16/32 (93) in APC, CD150 (mShad150) in PerCPCy5.5, cKit/CD117 (2B8) in SB 645, Sca-1 (D7) in PE-Cy7, CD48 (HM48-1) in AF700, Viability Dye in eFlour506.

Aortic tissue was stained subsequently: CD45 (30-F11) in APC-eFlour 780, CD90.2 (53-2.1) in brilliant violet 510TM, NK 1.1 (PK136) in PE-Cy7, F4/80 (BM8) in APC, CD11b (M1/70) in PE, Ly6G (1A8) in FITC, Ly6C (Al-21) in PerCP-Cy5.5 and Viability Dye in eFlour 506. Analysis was performed with FlowJo software (FlowJo Version 10, Treestar; Ashland, OR, USA).

### 2.8. mRNA Expression Analysis

A 7900HT Fast Real-Time PCR System (Applied Biosystems, Foster City, CA, USA) was used for mRNA expression analysis. Isolation of mRNA from frozen cardiac, splenic or aortic tissue was conducted by guanidine isothiocyanate phenol chloroform extraction. Real-time RT-PCR was performed with the CFX96 Real-Time PCR Detection System (Bio-Rad). Total mRNA (0.125 µg) was mixed with the QuantiTect Probe RT-PCR kit from Qiagen and probe and primer sets from TaqMan Gene Expression assays were applied (Applied Biosystems) for the following transcripts: CC-chemokine ligand2 (Ccl2; mouse: Mm00441242_m1), vascular cell adhesion molecule-1 (Vcam1, mouse:Mm00449197_m1), TATA-box-binding protein (mouse: Tbp, Mm00446973_m-1), interleukin 1 beta (Il1b; mouse: Mm00434228_m1), interleukin 6 (Il6; mouse:Mm00446190_m1), inducible nitric oxide synthase (iNOS, Nos2; mouse: Mm00440485_m1), endothelial nitric oxide synthase (eNOS, Nos3; mouse: Mm00435204_m1), CXC-motive-chemokine 12 (Cxcl-12; mouse: Mm00445553_m1), Angiopoetin-1 (Angpt-1; mouse: Mm00456503_m1), Kit-ligand/stem cell factor (Kitl; mouse: Mm00442972_m1), p47phox (mouse: Mm00447921_m1), tumor necrosis factor alpha (Tnf; mouse: Mm00443260_m1), angiotensin II receptor type 1 (Agtr1; mouse: Mm01957777_s1). The relative delta Ct method, normalized to TATA-box-binding protein as the endogenous control, was used for quantification and mRNA levels were expressed relative to levels of control.

### 2.9. Enhanced Chemiluminescence

Oxidative burst was measured in fresh citrate blood by L-012 (8-amino-5-chloro-7-phenylpyrido [3,4- d] pyridazine-1,4-(2H,3H) dione sodium salt, 100 µM) enhanced chemiluminescence (ECL) upon 1:50 dilution. Stimulation was performed with zymosan A (50 µg/mL) as well as phorbol ester dibutyrate (PDBu 10 µM) in PBS buffer containing Ca^2+^/Mg^2+^ (1 mM). Enhanced chemiluminescence (ECL) was analyzed using a TECAN plate reader (Tecan Group, Männersdorf, Switzerland).

### 2.10. Flourescence Oxidative Microtopography

Vascular ROS formation was analyzed with dihydroethidium (DHE, 1 µM)-dependent fluorescence microtopography of aortic cryo-sections as described. After preparation of aortic rings and incubation in Krebs-Hepes solution (15 min at 37 °C), they were embedded in OCT resin (TissueTek, Torrance, CA, USA) and frozen in liquid nitrogen. Cryosections of 8 µm were stained with dihydroethidin (DHE, 1 µM in PBS) and incubated for 30 min at 37 °C. Fluorescence (green autofluorescence, red DHE fluorescence) was measured with a Zeiss Axiovert 40 CFL microscope (Zeiss lenses LD A-plan 40×/o.50Ph2 and Axiocam MRm camera, Zeiss, Oberkochen, Germany).

### 2.11. Vascular Tone Experiments

To assess the vasodilative and vasoconstrictive characteristics of the vessels, 3-mm segments of the thoracic aortas were cut and mounted on force transducers (Kent Scientific Corporation, Torrington, CT, USA; Powerlab, ADInstruments, Spechbach, Germany) in organ chambers, which were filled with Krebs–Henseleit solution, and 10 µM indomethacin was added and gassed with carbogen. To test vascular function responding to acetylcholine (ACh) for testing endothelial-dependent and responding to nitrogylcerine (glycerol trinitrate, NTG) to test endothelial-independent vasorelaxation, aortic vessel rings were stretched gradually over 1 h to obtain tensions of 1.0 g. After preconstriction with phenylephrine (PHE) reaching 80% of maximal tone (induced by KCl), relaxation capability in responding to ACh or NTG was recorded as concentration-relaxation curves.

### 2.12. Statistical Analysis

Data are presented as mean ± standard error of the mean, except otherwise noted. To test whether data were normalized, they had to pass normality tests (D’Agostino or Kolmogorow–Smirnow test). When normal distribution was assumed, a t-test was performed to compare the two experimental groups. An ordinary one-way analysis of variance (ANOVA) followed by a Bonferroni test for multiple comparisons was used for multiple comparisons of more than two experimental groups. For comparison of more than two test groups or more than one analysis per group, a two-way ANOVA was applied. When the data were not normally distributed, two test groups were analyzed by t-test and Mann–Whitney U test. To compare more than two groups, a Kruskal–Wallis test was used, followed by Dunn’s test for multiple comparisons. Values of *p* < 0.05 were considered significant, marked by asterisks: * *p* < 0.05; ** *p* < 0.01; *** *p* < 0.001. To perform statistics, Version 8 of GraphPad Prism software (GraphPad Software Inc., La Jolla, CA, USA) was utilized.

## 3. Results

### 3.1. Immediate ACE Inhibition Post-MI Limits Infiltration of Inflammatory Monocytes in the Ischemic Myocardium due to Reduced Expression of Adhesion Molecules

**Figure 1:**
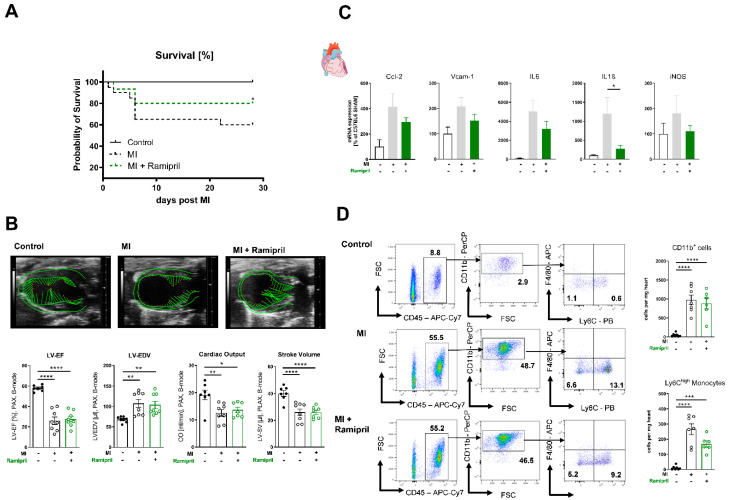
Early ramipril treatment limits infiltration of inflammatory monocytes to the ischemic myocardium and expression of adhesion molecules after MI(myocardial infarction). (**A**) Kaplan–Meier survival curve after MI or MI mice with ramipril treatment vs. sham-treated (control) over the period of 28 days. n = 12–26 per group; Log-Rank (Mantel–Cox test). (**B**) Transthoracic echocardiography measured in parasternal long axis (PLAX) with analysis of left ventricular ejection fraction (LV-EF), left ventricular end-diastolic diameter (LV-EDV), cardiac output and stroke volume on day 6 after MI vs. sham, (top) representative PLAX B-mode images, (bottom) quantification; n = 7–8 per group; (**C**) mRNA expression of heart tissue of Ccl-2, Vcam-1, Il6, Il1b and iNOS (Nos2) 7 days after MI and sham operation; n = 6–7 mice per group; (**D**) Left: Representative gating strategies of CD45^+^CD3^−^CD11b^+^ and CD45^+^CD3^−^CD11b^+^Ly6G^−^Ly6C^high^. Bold numbers indicate the percentual ratio of total living cells. Right: Flow cytometry quantification of infiltrating CD11b^+^ myeloid cells and Ly6C^high^ monocytes in the infarcted heart vs. sham operation 7 days after MI, n = 6–7 mice per group; mean + SEM, 1-way ANOVA or Kruskal–Wallis test with Dunn’s multiple comparisons test, * *p* < 0.05, ** *p* < 0.01, **** p < 0.0001.

Early administration of an ACE inhibitor with consecutive RAS blockade improves overall survival in ischemic heart failure (Figure 1A), without significantly altering left ventricular function within 6 days after MI. (Figure 1B). The mRNA expression of myeloid cell adhesion molecules such as CC-chemokine ligand2 (*Ccl2*) or vascular cell adhesion molecule-1 (*Vcam-1*) was reduced in heart tissue, but did not reach significance. Furthermore, we detected reduced mRNA levels of pro-inflammatory chemokines such as interleukin 6 (*Il6*) or 1 beta (*Il1ß*) in infarcted myocardium after ramipril treatment (Figure 1C). In order to investigate the beneficial effects of ramipril treatment in more detail, we performed flow cytometric analysis of infarcted myocardium 7 days after MI. In line with the diminished myocardial expression of *Ccl2*, *Vcam-1*, *Il6* and *Il1ß* in treated animals, we revealed an accumulation of myeloid cells, especially inflammatory Ly6C^high^ monocytes, into the infarcted myocardium, which was reduced by the trend in mice treated with the ACE inhibitor (Figure 1D).

### 3.2. Ramipril Limits the Number of Circulating Monocytes and Retains HPSC Due to Upregulation of Retention Factors in the Bone Marrow and Spleen

**Figure 2:**
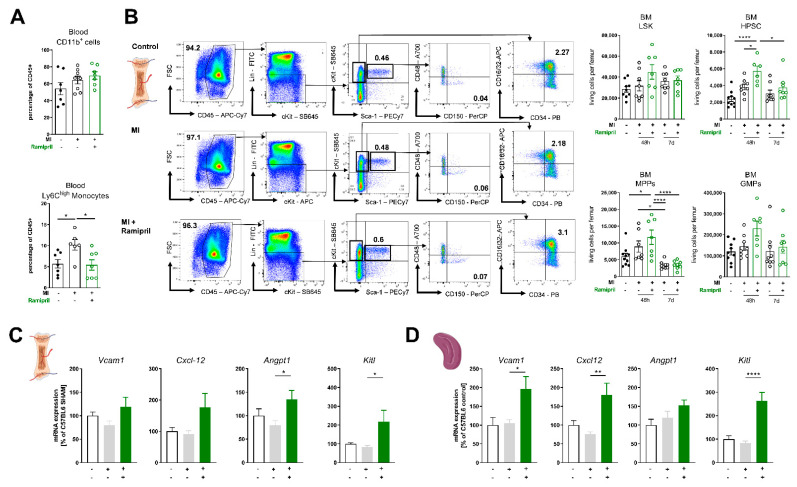
ACE (angiotensin-converting-enzyme) inhibition limits the number of circulating monocytes and retains HPSC (hematopoetic stem cells) due to upregulation of retention factors in the bone marrow and spleen. (**A**) Flow cytometry quantification of circulating CD11b^+^ myeloid cells and Ly6C^high^ monocytes in whole blood 7 days after MI, n = 6–7 mice per group; (**B**) Quantification of LSK, HPSCs, MPPs and GMPs in the bone marrow 48 h and 7 d post-MI; left: representative FACS (fluorescent activated cell sorting) plots; bold numbers indicate percentual ratio of total living cells, right: quantification of Lineage^−^/Sca1^+^/cKit^+^ LSK, Lineage^−^/Sca1^+^/cKit^+^/CD48^−^/CD150^+^ HPSCs, Lineage^−^Sca1^−^/cKit^+^/CD48^−^/CD150^−^ MPPs, Lineage^−^Sca1^−^/cKit^+^/CD16/CD32^+^/CD34^+^ GMPs per femur; n = 7–10, (**C**) + (**D**) mRNA expression of Vcam-1, Cxcl-12, Angpt1 and Kitl in the bone marrow (**C**) and spleen 7 days after MI (**D**), n = 4–8 mice per group; mean + SEM, 1-way-ANOVA or Kruskal–Wallis test with Dunn’s multiple comparisons test. * *p* < 0.05, ** *p* < 0.01, **** *p* < 0.0001.

AngII signaling is crucial post-MI, and administration of AngII causes an intense mobilization of HPSC [10]. We therefore investigated how lowering of AngII levels due to ACE inhibition impacts emergency myelopoiesis in cardiac ischemia. Circulating levels of CD11b^+^ myeloid cells were increased after MI and were not affected by ACE-I treatment; interestingly, the number of circulating Ly6C^high^ monocytes was statistically significantly lower in the treatment group post-MI (Figure 2A). It has been shown that cardiac ischemia stimulates the production and release of HPSC. Early and rapid leukocytosis is typical post-MI, whereas most of these cells are part of the innate immune system and derive from myeloid origin [3]. Furthermore, 48 h post-MI, we analyzed the bone marrow and detected an increased number of CD150^+^CD48^−^ pluripotent hematopoietic stem cells, Lin^−^Sca-1^−^c-Kit^+^CD34^+^CD16/32^+^ granulocyte–macrophage progenitors and Lin^−^Sca-1^−^c-Kit^+^CD150^−^CD48^−^ multipotent progenitors. This effect was even more pronounced in response to ramipril treatment. The amount of precursor cells in the bone marrow normalized over time and we did not detect a significant difference between the MI groups with or without ACE-I treatment at 7 d post-MI (Figure 2B). The proliferation of HPSC and release of mature leukocytes is regulated by the hematopoietic niche and proteins encoded by genes such as *angpt1*, *kitl*, *Vcam1* or *Cxcl-12* [14,15]. Expression of these genes was significantly higher in mice with ACE inhibitor treatment 7 d post-MI in the bone marrow and spleen (Figure 2C,D). In summary, we noticed an extended myeloid hematopoiesis as a response to myocardial ischemia. ACE inhibition increases the number of progenitor cells in the bone marrow but reduces the number of circulating inflammatory myeloid cells. As a potential mechanism, we found enhanced expression of retention factors in the bone marrow niche and spleen.

### 3.3. Early ACE Inhibitor Treatment Improves Vascular Endothelial Function and Leads to Attenuation of Vascular ROS and Inflammation in Ischemic Heart Failure

**Figure 3:**
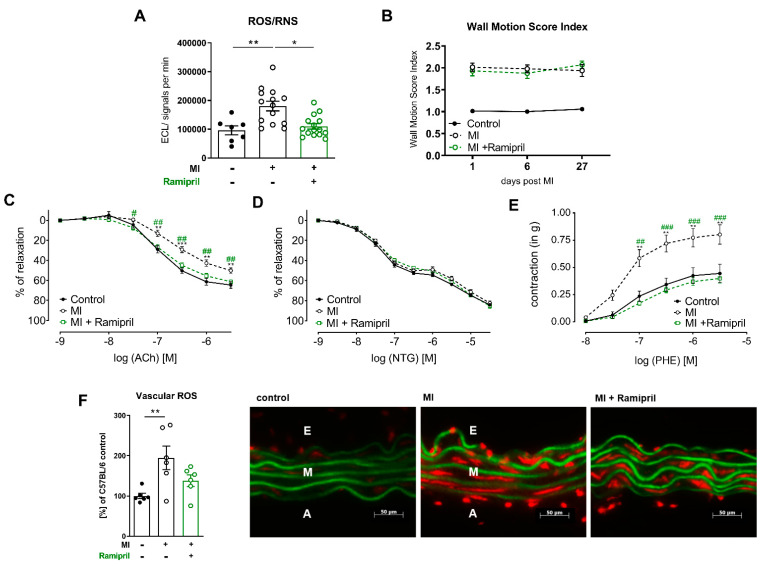
In ischemic heart failure, early ACE inhibitor treatment improves vascular endothelial function due to reduction of vascular and systemic ROS. (**A**) Superoxide formation in whole blood measured by enhanced chemiluminescence after stimulation with PDBu 28 d after MI, n = 7–15 mice per group; (**B**) High-frequency ultrasound echocardiography with measurement of wall motion score index (WMSI) 4 weeks post-MI, n = 13 to 14 mice per group; (**C**–**E**) Isometric tension studies of isolated aortic segments 28 d after MI. (**C**) Contraction-relaxation curves in response to endothelium-dependent vasodilator acetylcholine (Ach), (**D**) contraction-relaxation curves in response to endothelium-independent vasodilator nitroglycerin (NTG), (**E**) dose-dependent contraction curve in response to the α1-agonist phenylephrine (PHE); n = 4–12 rings per group; mean + SEM; 2-way ANOVA; * *p* < 0.05, ** *p* < 0.01 (Control vs. MI), # *p* < 0.05, ## *p* < 0.01, ### *p* < 0.001 (MI vs. MI + ramipril); (**F**) Oxidative fluorescence microtopography; right: per group, one representative photomicrograph is shown. Autofluorescence of laminae produces a green signal, superoxide formation yields a red fluorescent signal; A, adventitial layer; E, endothelial layer; M, medial layer; left: quantification of superoxide formation in the vessel wall measured by integrated optical density (IOD), percentage of C57BL6 sham; n = 6 per group; mean + SEM; one-way ANOVA. *p* < 0.05, ** *p* < 0.01, *** *p* < 0.001.

**Figure 4:**
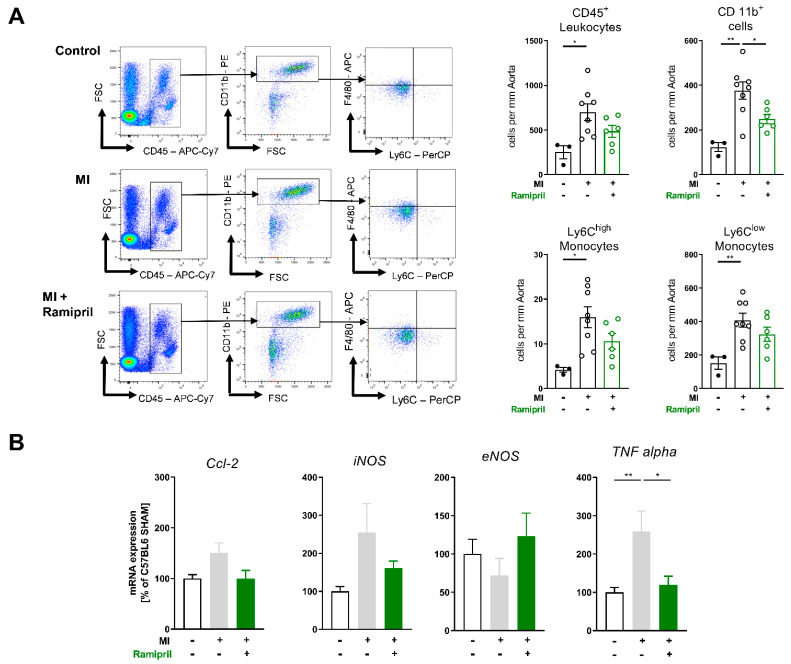
Ramipril limits vascular inflammation in chronic ischemic heart failure by attenuating myeloid cell infiltration. (**A**) Flow cytometry quantification of aortic tissue 28 days after MI vs. sham; left: representative FACS plots, right: quantification of leucocytes (CD45^+)^, myeloid cells (CD45^+^CD11b^+^) and monocytes (CD45^+^CD11b^+^Ly6G-Ly6C^high^/^low)^, n = 3–8 mice per group, mean + SEM, n = 3–8 mice per group; (**B**) mRNA expression of aortic tissue of Ccl-2, iNOS (Nos2), eNOS and TNFalpha 28 days after MI vs. sham (+Ramipril); mean + SEM, 1-way ANOVA or Kruskal–Wallis test with Dunn’s multiple comparisons test. * *p* < 0.05, ** *p* < 0.01.

We could recently show that depletion of myeloid cells as well as ARB treatment improves vascular endothelial function and survival in a disease model of ischemic heart failure. In line with this, in the current study, ACE inhibitors improved long-term survival post-MI as well (Figure 1A). Therefore, we investigated the effects of early ramipril treatment after permanent LAD ligation for 28 days on the vascular homeostasis, inflammation and ROS production. ACE inhibition limited the production of reactive oxygen species in whole blood assessed by L-012 enhanced chemiluminescence (ECL) stimulated with PDBu (Figure 3A). Echocardiographic examination and quantification of infarct size by wall motion score index over 4 weeks showed no difference between the MI- and ACE-inhibitor-treated groups (Figure 3B). Mice with ischemic heart failure developed vascular endothelial dysfunction, confirmed by organ chamber experiments with isolated aortic rings in response to the vasodilator acetylcholine (ACh). Endothelial dysfunction post-MI was attenuated in response to 28-d treatment with ramipril (Figure 3C). Endothelial-independent vascular relaxation in response to glycerol trinitrate (nitroglycerin, NTG) was not affected. Vessels from MI mice also showed enhanced sensitivity to adrenergic stimulation with the alpha1 receptor agonist phenylephrine (PHE) (Figure 3D,E). Neurohumoral activation involving both increased sympathetic outflow and RAAS activation is characteristic of heart failure post-MI, and angiotensin II is a potent stimulator of vascular ROS sources such as NADPH oxidase Nox1 and Nox 2 [16,17]. In line with this, the production of vascular ROS measured by fluorescence oxidative microtopography was increased in heart failure mice and improved with ACE inhibition (Figure 3F). Flow cytometry analysis of aortic tissue showed an attenuated infiltration of myeloid cells, especially Ly6C^high^ and Ly6C^low^ monocytes, into the vessel wall (Figure 4A). In line with the diminished myocardial infiltration of myeloid cells in treated animals, the mRNA expression of the chemokine TNFα was significantly reduced and the adhesion molecules CC-chemokine ligand2 (Ccl2) as well as the inducible NO-synthase (iNOS) were lowered by this trend (Figure 4B).

## 4. Discussion

Cardiac ischemia is followed by an excessive immune response in order to rescue the damaged tissue. Dying cells release an amount of pro-inflammatory cytokines and mediators such as TNFα, interleukin 6 and 1 as well as interferon *γ*, which not only act locally but also circulate and stimulate HPSC to trigger the production of innate immune cells [12,18,19,20]. Such external alarm signals activate intracellular signal cascades and induce the production of multiple transcription factors such as PU.1 or Egr-1 and activate HPSC [2,21]. On the other hand, it could be shown that they lead to reduced expression of bone marrow niche factors such as Cxcl-12, Vcam-1 or Kitl to mobilize innate immune cells to the blood circulation [22,23,24]. These mechanisms are in part controlled by sympathetic activation due to ß3 adrenergic receptors [22]. The effects of other hormones, particularly AngII, in this process were incompletely understood. In the current study, we were able to present new insights into AngII interactions with the innate immune system after MI, which could be diminished by pharmacological blockade of AngII synthesis. We could show that ACE inhibition by ramipril limits the number of circulating Ly6C^high^ monocytes one week after MI. We could also demonstrate upregulation of retention factors and higher amounts of myeloid progenitor cells in the bone marrow and spleen in ramipril-treated animals 48 h after MI, indicating increased retention of mature myeloid cells in the bone marrow. Studies in the past showed that ACE inhibitors attenuate myeloid tissue infiltration by reducing levels of monocyte chemoattractant protein-1 (MCP-1). Moreover, limited expression of vascular cell adhesion molecule-1 in hematopoietic niches (VCAM-1) could be shown [25,26].

After survival of an acute MI, there is still a high risk for recurrent cardiovascular events. Within the first year, the frequency of a secondary MI, stroke or cardiovascular death is 8–12%, despite optimal medical treatment [27,28,29]. A meta-analysis concluded that early administration of ACE inhibitors within 72 h to 14 days post-MI lowers the risk of further cardiovascular events and improves survival in humans, as the SAVE trial demonstrated as well [30,31,32]. Exact underlying mechanisms are still incompletely understood and probably manifold, but with our data, we could indicate suppressed systemic inflammation with pharmacological ACE inhibition. In the long-term experiments, ACE inhibition showed less accumulation of inflammatory myeloid cells in the vascular wall and less NADPH-oxidase-derived reactive oxygen species. Attenuated vascular oxidative stress indicated beneficial effects on endothelial function, which we analyzed in isometric tension studies.

The expression of ACE is also significantly increased at the edge of infarcted scars and it is well known that AngII, as the effector hormone, is crucial in the cardiac remodeling process after MI [33]. In line with this, we could detect less myeloid infiltration of ischemic cardiac tissue and less expression of inflammatory cytokines seven days after MI in ramipril-treated mice, although the results were not statistically significant. Significant effects on cardiac function within four weeks of treatment could be excluded in our study, which was underlined by unaltered echocardiographic findings in the different groups. Besides the reduction of cardiac afterload via endothelial AngII type 1 receptors, there are more mechanisms contributing to the pharmacological effects of RAS blockade. ACE inhibitors were shown to improve the oxygen supply/demand ratio of the myocardium by attenuating AngII-induced vasoconstriction and inotropic activity, increasing the endothelial ability to relax vascular smooth muscles.

Moreover, there is growing evidence that an MI and consecutive ischemic heart disease leads to persistent systemic inflammation. In this context, it has been shown that patients with higher inflammatory burden have an elevated risk for additional cardiovascular events [20,34]. Treating long-lasting inflammation is at the center of current cardiovascular research [35,36]. Currently, it is unclear whether current therapies are able to tackle these effects, so we tried to elucidate the pleiotropic effects of RAS inhibition after cardiac ischemia [3,37]. In past studies by our group, we already showed the strong effects of ARB treatment on persistent systemic inflammation four weeks after MI, which were comparable with the current study in improving vascular function. Although we did not analyze acute myelopoiesis after MI, we could show even stronger effects on suppressing vascular infiltration by myeloid cells 28 d after MI.

## 5. Limitations of the Study 

Treatment with the ACE inhibitor ramipril followed already published protocols, but was not verified in our study by blood pressure recordings or by measurements of circulating AngII levels. Nevertheless, we detected effects on systemic and tissue-specific inflammation, which led us to conclude that our treatment has been effective. In some experiments, the number of used animals was low and our results failed to reach statistical significance. The corresponding results are indicated in the results section and the interpretation of the data should be carried out with careful consideration. Furthermore, the expression of the cytokines, adhesion molecules and retention factors was only verified by real-time PCR analysis at the mRNA level. Conformation of the protein levels was not performed.

## 6. Conclusions

Taken together, our findings allow us to conclude that, besides the well-known beneficial effects on cardiac remodeling and blood pressure control, there are additional effects of RAS inhibition on emergency myelopoiesis after MI, resulting in less long-lasting systemic inflammation and improving endothelial function and survival without enhancing cardiac function.

## Data Availability

The data presented in this study are available on request from the corresponding author.
